# Validation of Computerized Automatic Calculation of the Sequential Organ Failure Assessment Score

**DOI:** 10.1155/2013/975672

**Published:** 2013-07-09

**Authors:** Andrew M. Harrison, Hemang Yadav, Brian W. Pickering, Rodrigo Cartin-Ceba, Vitaly Herasevich

**Affiliations:** ^1^Medical Scientist Training Program, Mayo Clinic, 200 1st Street SW, Rochester, MN 55905, USA; ^2^Multidisciplinary Epidemiology and Translational Research in Intensive Care (METRIC), Mayo Clinic, 200 1st Street SW, Rochester, MN 55905, USA; ^3^Department of Medicine, Mayo Clinic, 200 1st Street SW, Rochester, MN 55905, USA; ^4^Department of Anesthesiology, Mayo Clinic, 200 1st Street SW, Rochester, MN 55905, USA; ^5^Division of Pulmonary and Critical Care Medicine, Department of Medicine, Mayo Clinic, 200 1st Street SW, Rochester, MN 55905, USA

## Abstract

*Purpose*. To validate the use of a computer program for the automatic calculation of the sequential organ failure assessment (SOFA) score, as compared to the gold standard of manual chart review. *Materials and Methods*. Adult admissions (age > 18 years) to the medical ICU with a length of stay greater than 24 hours were studied in the setting of an academic tertiary referral center. A retrospective cross-sectional analysis was performed using a derivation cohort to compare automatic calculation of the SOFA score to the gold standard of manual chart review. After critical appraisal of sources of disagreement, another analysis was performed using an independent validation cohort. Then, a prospective observational analysis was performed using an implementation of this computer program in AWARE Dashboard, which is an existing real-time patient EMR system for use in the ICU. *Results*. Good agreement between the manual and automatic SOFA calculations was observed for both the derivation (*N=94*) and validation (*N=268*) cohorts: 0.02 ± 2.33 and 0.29 ± 1.75 points, respectively. These results were validated in AWARE (*N=60*). *Conclusion*. This EMR-based automatic tool accurately calculates SOFA scores and can facilitate ICU decisions without the need for manual data collection. This tool can also be employed in a real-time electronic environment.

## 1. Introduction

Interest in assessment of organ dysfunction and severity of illness in the intensive care unit (ICU) setting has increased in recent years. The acute physiology and chronic health evaluation (APACHE) is one of the earliest scoring systems developed for this purpose and is currently in its fourth iteration [1, 2]. Other scoring systems have been developed, including the simplified acute physiology score (SAPS) and the mortality probability model (MPM) [3, 4]. The sequential organ failure assessment (SOFA) score is a 24-point scale designed to assess organ dysfunction and failure in critically ill patients [5]. SOFA is frequently used for the assessment of severity of illness in the ICU setting and is based on a six component, organ-based scoring system [6]. SOFA has many advantages over previously developed systems, including simplicity and ease of calibration. Traditionally, manual calculation of these scores—including SOFA—has been a time-consuming and error prone task. However, the ability of automatic tools to be used for risk assessment of patients has been demonstrated by multiple studies [7, 8].

Several automatic SOFA calculation tools have been developed. One of the earliest such tools was created and validated by Junger et al. [9]. However, it was necessary to modify the validated SOFA scoring system to allow automated calculation. Engel et al. demonstrated that a computerized calculation of derived SOFA measures correlated with length of stay [10]. However, a validation of this tool against the gold standard of manual chart review was lacking. As another example, Nates et al. developed a tool for automatic calculation of a modified SOFA score [11] and used this system for modeling of ICU and/or hospital mortality in cancer patients [12]. Although additional automated SOFA scoring tools have been recently developed, detailed methodology of the validation of these tools is lacking [13, 14]. Therefore, a clear need is present for the development of a fully automatic SOFA tool and its validation against the gold standard of manual chart review. The objective of this study was to develop, validate, and implement a modern EMR computerized automatic SOFA calculator and compare it against the gold standard of manual chart review and calculation.

## 2. Materials and Methods

### 2.1. Study Design and Settings

Retrospective cross-sectional analysis of consecutive adult admissions (age > 18 years) to the medical ICU with a length of stay greater than 24 hours was performed. For the derivation cohort, these admissions were from January 2007 through January 2009. For the validation cohort, these admissions were from February 2011 through March 2011. For both cohorts, automatic calculation of the SOFA score was compared to the gold standard of manual chart review and followed by critical appraisal of sources of disagreement. For the prospective observational analysis, we studied all admissions to medical ICU during six days. Patients who denied research authorization were excluded. The Institutional Review Board approved the study protocol and waived the need for informed consent.

### 2.2. Electronic Resources

The Critical Care Independent Multidisciplinary Program (IMP) of the study hospital has an established near-real-time relational database, the Multidisciplinary Epidemiology and Translational Research in Intensive Care (METRIC) Data Mart. Details of METRIC Data Mart and the EMR are previously published [15]. METRIC Data Mart contains a near-real-time copy of patient's physiologic monitoring data, medication orders, laboratory and radiologic investigations, physician and nursing notes, respiratory therapy data, and so forth. Access to the database is accomplished through open database connectivity (ODBC). Blumenthal et al. [16] and Jha et al. [17] have reached consensus through Delphi survey on defining a comprehensive EMR, which requires 24 key functions to be present in all the clinical units of hospital. The current Mayo Clinic EMR fits the definition of a comprehensive EMR. Upon request, we are happy to provide detailed information regarding our algorithms and system.

### 2.3. SOFA Score Description

SOFA is a scoring system based on six organ components: respiration, coagulation, liver, cardiovascular, central nervous system, and renal. Each component is assigned a severity score from 0 to 4, with 24 being the maximum possible severity score total [5]. The respective component scores are based on calculation of PaO_2_/FiO_2_ and respiratory support, platelets, bilirubin, mean arterial pressure and adrenergic agent administration, Glasgow coma score, and creatinine and urine output. When multiple values of SOFA elements were present within a single day for a patient record, the highest value was recorded.

### 2.4. Gold Standard Development

The gold standard of manual chart review for the derivation and validation cohorts was performed by evaluation of EMR-based patient records over the first 24 hours of admission by a trained coinvestigator (AMH).

### 2.5. Derivation and Validation Cohort Descriptions

The derivation analysis was performed with a cohort of 94 patients. The validation of the analysis was performed with a cohort of 268 patients.

### 2.6. Other Study Procedures

Critical appraisal of sources of disagreement between these scores was performed to allow for subsequent adjustment of the automatic tool for use on the validation dataset. To perform this analysis, records for which the disagreement between automatic and manual calculation was greater than two were selected. The automatic and manual calculations for these records were then reviewed manually by organ system to determine the source of error. The results of this analysis were used to identify common sources of error for modification of subsequent dataset calculations.

### 2.7. AWARE Validation Description

AWARE (Ambient Warning and Response Evaluation) Dashboard is an ICU rule-based patient viewer and electronic-environment enhancement program, which extracts and presents patient information that is relevant to the ICU setting from the standard EMR [18]. This information is grouped by organ system, color-coded by degree of disease severity, and displayed as an easy-to-read, one-page interface on a monitor display. In comparison to the standard EMR, AWARE has been demonstrated to reduce task load and errors in a simulated clinical experiment [19]. The medical charts of 60 patients in the medical ICU at Mayo Clinic were reviewed over the course of six days. For visual display purposes, AWARE automatically calculates and presents the six SOFA organ system subscores using a scaled, three-tiered scoring system with color-coded icons: white (SOFA = 0 or 1), yellow (SOFA = 2), or red (SOFA = 3 or 4). At the same time, the EMR was used for the manual calculation of real-time scores as the gold standard. For the purpose of this analysis, manual SOFA scores were subsequently converted into the three-tiered scoring system that has been implemented in AWARE. Manual calculation of the SOFA score was performed by a trained reviewer (AMH). Results were analyzed using JMP statistical software (SAS Institute Inc).

### 2.8. Outcomes

The primary outcome measured in this study was agreement between SOFA scores calculated using the gold standard of manual chart review and the automatic tool. Agreement was determined using Bland-Altman analysis. Critical appraisal of sources of disagreement for individual SOFA components was also performed.

### 2.9. Statistical Considerations

The agreement in calculated SOFA between automatic and gold standard calculation is reported using correlation coefficient, and the difference is reported with SD. Comparison and analysis of these calculations were performed using Bland-Altman plots [20]. This method of comparative analysis is used to assess agreement between two methods of measurement, especially when a gold standard exists [21]. It has been used previously to compare variability in ICU scoring systems, including APACHE, SAPS, and SOFA [22–24]. For baseline characteristics (Table 1) and Wilcoxon signed-rank testing, two-sided significance testing was performed with a *P* value less than 0.05 considered significant. All the data analyses were performed using JMP (SAS, Cary, NC) statistical software. The results of this analysis have been reviewed by a Mayo Clinic statistician.

## 3. Results

### 3.1. Retrospective Cross-Sectional Analysis: Automatic Tool Development

**Figure 1:**
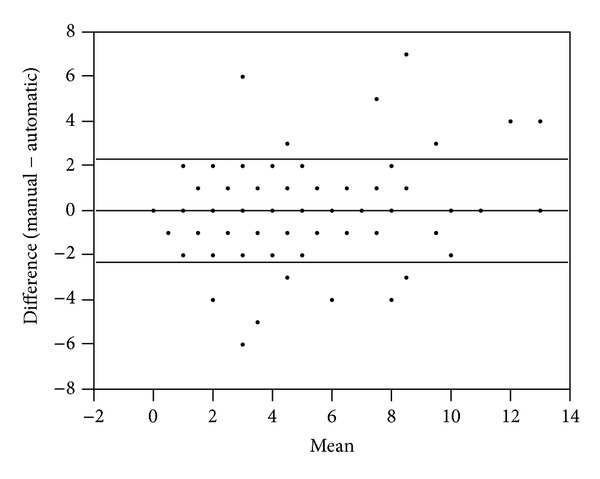
Derivation cohort: Bland-Altman plot of the manual versus automatic scores resulted in a mean difference of 0.02 ± 2.33 (SD, *N* = 94). Note: plotted values frequently represent more than one patient sample.

**Figure 2:**
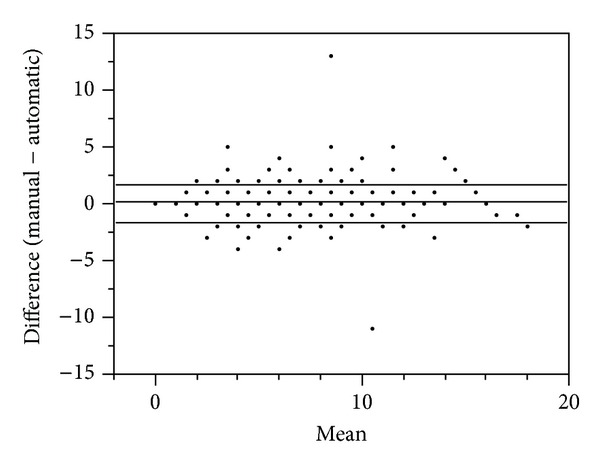
Validation cohort: Bland-Altman plot of the manual versus automatic scores resulted in a mean difference of 0.12 ± 1.64 (SD, *N* = 268). Note: plotted values frequently represent more than one patient sample.

There was no statistically significant difference in the derivation and validation cohorts with respect to sex or frequency of low SOFA scores (Table 1). However, there was a statistically significant difference between these cohorts with respect to age and mean ICU length of stay (Table 1). Comparison of the automatic and manual SOFA calculations in both the derivation and validation datasets was approached through use of Bland-Altman plots. In the case of the derivation dataset, Bland-Altman plotting of the difference between scores versus the mean of these scores resulted in a mean difference of 0.02 ± 2.33 (Figure 1). In the case of the validation dataset, an identical Bland-Altman approach resulted in a mean difference of 0.29 ± 1.75 (Figure 2). Thus, although there was a slight increase in deviance of the mean difference from zero in the validation dataset, as compared to the derivation dataset, a decrease in the respective standard deviation was observed.

**Figure 3:**
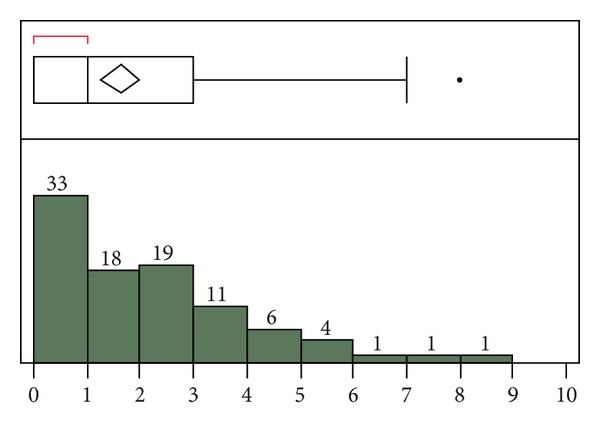
Derivation cohort: sum of the absolute value of the difference between the automatic and manual SOFA component scores. For the outlier box plot, the first box (red bracket) represents the first quartile, followed by the second and third quartiles. Fourth quartile outliers are represented by black dots. The triangle represents the mean difference (1.64).

**Figure 4:**
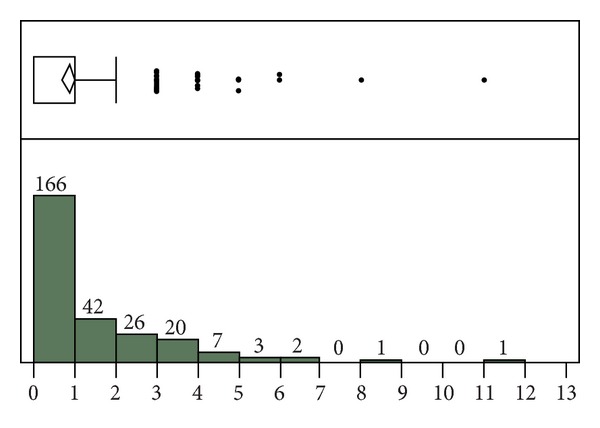
Validation cohort: sum of the absolute value of the difference between the automatic and manual SOFA component scores. For the outlier box plot, the first box represents the first and second quartiles, followed by the third quartile. Fourth quartile outliers are represented by black dots. The triangle represents the mean difference (0.85).

Bland-Altman plotting can obscure cases of disagreement where large deviations in both directions result in averages close to zero. To better represent these cases, calculation of the sum of the absolute value of the difference between the automatic and manual SOFA component scores was performed. For the derivation dataset, the median for the sum of absolute value of this difference was 1 (Figure 3). The mean difference was 1.64 for this dataset. For the validation dataset, these values were 1 and 0.85, respectively (Figure 4). Thus, as with the Bland-Altman plot, the agreement between the automatic and manual scores was improved in the validation dataset. Because averaging large deviations in both directions can also introduce differences into the mean rank of the population distribution, nonparametric testing was performed, using the Wilcoxon signed-rank test. However, this analysis revealed no statistically significant difference for the derivation (*P* = 0.85) or validation (*P* = 0.22) dataset. Thus, it was necessary to perform critical assessment of outliers to assess sources of significant discrepancy.

To better understand the deviation between the automatic and manual scores in both datasets, critical appraisal of differences was performed. In the case of the derivation dataset, 20% of the total SOFA scores were found to disagree by more than 2 points (Table 2). With respect to these cases of greatest disagreement, the automatic tool failed to compute 23% of all component scores, with the hepatic score being the most frequently omitted. However, as these missing scores account for, at most, only 34% of the total disagreement between the automatic and manual scores, this cannot fully explain all discrepancies. In fact, the majority of all disagreements could be found in the respiratory (34%) and renal (28%) components.

Likely sources of disagreement were first sought in the respiratory and renal components. This investigation revealed failure of the automatic tool to recognize patient ventilator status as one likely source of error. Another likely source of error was in the failure of the automatic tool to accurately factor scenarios of decreased urine output.

In the case of the validation dataset, 12% of the total SOFA scores were found to disagree by more than 2 points (Table 3). It was also found that the automatic tool failed to compute all component scores in 12% of these cases of greatest disagreement. Once again, the hepatic score was omitted most frequently. As these missing scores account for, at most, 7% of the total disagreement between the automatic and manual scores, this once again cannot fully explain all discrepancies. In this case, the majority of all disagreements could be found in the CNS component (61%). The primary sources of this error are programming and timing issues related to the automatic calculation of this component of the SOFA score, which must be obtained and entered manually into the EMR.

For the derivation dataset, the difference between the total automatic and manual SOFA scores for any sample did not disagree by more than seven points (Figure 2). For the validation dataset, only two samples disagreed by more than five points (Figure 3). For the case of greatest disagreement, the manual score exceeded the automatic score by 13 points. In agreement with the discrepancies described above, this disagreement was effectively a worst case scenario and caused by an underestimation by the automatic tool in five out of six component scores. For the case of second greatest disagreement, the automatic score exceeded the manual score by 11 points. This disagreement was caused by manual scorer error in calculation of the SOFA score during an incorrect 24-hour time period. Upon manual recalculation, the disagreement between these scores was reduced to zero.

### 3.2. Prospective Observational Analysis: Automatic Tool Validation in AWARE

We found good overall agreement between automatic calculation of the SOFA score in AWARE versus the gold standard of manual chart review for 60 patients (Table 4). Good agreement was found for the coagulation, liver, cardiovascular, renal, and central nervous components of the score, while the respiratory score had a higher level of disagreement. Further investigation determined the source of the majority of this error to be due to a failure of the automatic rule to assign a respiratory score of at least 3 to patients who were mechanically ventilated, resulting in an underestimation of the automatic score. 

## 4. Discussion

The primary purpose of this study was to validate the use of a computer program for the automatic calculation of the SOFA score. For the retrospective cross-sectional analysis, good agreement between the automatic and manual scores with respect to mean difference and standard deviation was observed for both the derivation and validation cohorts. Agreement of the automatic tool with the gold standard of manual chart was increased in the validation cohort after critical appraisal of sources of disagreement and modification of the original algorithm. The success of this approach is supported by the observation that there is a decrease in the standard deviation of the mean SOFA score in the larger validation cohort as compared to the smaller derivation cohort. Similarly, the rate of disagreement between the manual and automatic SOFA calculations is decreased in the validation cohort as compared to the derivation cohort. For the prospective observational analysis, this SOFA automatic calculator was validated within AWARE.

The literature reports several attempts to develop and implement automatic SOFA tools. One of the earliest studies to do this used a modified SOFA scoring system to retrospectively examine patients in the operative ICU [9]. Using an automatic SOFA tool and mortality as a functional outcome, the authors demonstrated that the mean modified SOFA score of patients who survived their ICU stay was higher than that of patients who did not survive. Although this study demonstrated the feasibility of using such an automatic SOFA tool to analyze functional outcomes in a large patient population, it did not compare these results to a gold standard of manual chart review. Thus, it is difficult to assess the accuracy of the results obtained. Using similar methodology and the same dataset, this group has since gone on to demonstrate that derivable measures from this tool, such as maximum SOFA score, are better predictors of mean length of stay than SOFA score upon admission [10]. As with the original study, these results were not compared to the gold standard of manual chart review.

Other groups have independently developed and implemented additional automatic SOFA tools toward similar goals. One such example is a recent, large retrospective study of several thousand cancer patients in the medical and surgical ICU of a single institution over the course of one year [12]. As with the previously described studies, the feasibility of using an automatic SOFA tool to analyze functional outcomes was demonstrated. However, the accuracy of the results obtained, as compared to the gold standard of manual chart review, was not demonstrated. Using similar methodology, this group had previously demonstrated the ability of their tool to calculate mean admission SOFA scores [11]. Thus, the feasibility of this approach has been demonstrated by at least two independent groups.

Several studies exist which have attempted to develop and validate automatic SOFA tools against the gold standard of manual chart review. One such example is a recent, large prospective study by another independent group [13]. The purpose of this study was to compare the performance of the SOFA score against another validated organ dysfunction scoring system, termed MOD (multiple organ dysfunction), for which another automatic tool was developed. However, this study provides no detailed description of the automatic validation process against the gold standard of manual chart review. Thus, it is once again difficult to assess the accuracy of these results. It is also worth noting that another independent group has recently published a correspondence in which 50 SOFA scores are stated to have been computed automatically and then validated against the gold standard of chart review [14]. However, as this data is not included in the correspondence, it is not possible to comment further.

### 4.1. Limitations

The current study has the limitation of calculation of the gold standard of manual chart review by a single reviewer in the setting of a single ICU. However, our study is strengthened by the completeness of the data supported by electronic infrastructure. Further validation of this automatic tool will require multiple-ICU studies. An important long-term consideration in designing these studies is compatibility between EMR systems across multiple institutions [25]. The challenge of systems compatibility between hospitals for long-term and implementation studies is amplified by the presence of multiple major EMR vendors, as well as custom systems [26]. Effectively, this challenge may result in differences in the availability of this tool in different ICU settings. Furthermore, differences across multiple institutions in the extent to which EMR data entry is automated can influence the degree of human error introduced into this process.

Another limitation of this study is the accuracy of the automatic tool. Cases of missing automatic hepatic scores were identified when this data was available in the EMR. There were also failures of the automatic tool to recognize correct ventilator status, calculate correct urine output, and extract CNS scores at the correct time. Critical appraisal of the source of these errors in the derivation dataset was demonstrated to result in improved calculation in the validation dataset. However, some errors still persisted. Additionally, similar errors were observed in the prospective observational analysis using the real-time AWARE patient monitoring system.

## 5. Conclusion

This study validates the use of a computer program for the automatic calculation of the SOFA score as compared to the gold standard of manual chart review. This automatic tool has the potential to be further developed and improved. However, the present study validates the use of this automatic calculator in the EMR-viewer AWARE for visualization and real-time SOFA calculation in the ICU. As demonstrated using AWARE, this program allows automatic and rapid calculation of SOFA scores in hospitalized patients. Fully implemented, this tool may be useful when utilizing the SOFA score to assess the presence and extent of organ dysfunction. However, testing this hypothesis will require an investigation of its prognostic value in a cohort of ICU patients.

## Figures and Tables

**Table 1:** Baseline characteristics of the derivation and validation cohorts.

Variable	Derivation cohort (*N* = 94)	Validation cohort (*N* = 268)	*P* Value
Age (years), mean ± SD	54.7 ± 13.8	65.6 ± 17.6	<0.0001
Sex, male (%)	53 (56)	154 (57)	0.86
ICU length of stay, mean ± SD	2.8 ± 3.2	3.8 ± 4.1	0.03
Low SOFA score (<6)	50%	48%	0.29

**Table 2:** Derivation cohort subset: difference (manual score − automatic score) greater than two. Difference in component scores is also included. Total represents the sum of the absolute value of the differences.

Patient	Diff	Resp	Coag	Liver	CV	CNS	Renal
Derv 1	7	−1	1	2	1	0	4
Derv 2	7	3	0	3	0	0	1
Derv 3	6	2	0	0	0	0	4
Derv 4	5	2	0	3	0	0	0
Derv 5	4	0	4	0	0	0	0
Derv 6	4	0	−1	0	3	3	−1
Derv 7	3	2	0	0	1	0	0
Derv 8	3	3	0	0	0	0	0
Derv 9	3	1	0	0	1	1	0
Derv 10	−3	−2	0	0	0	−1	0
Derv 11	−3	0	0	0	0	0	−3
Derv 12	−3	−2	−1	0	0	0	0
Derv 13	−3	0	0	0	0	0	−3
Derv 14	−4	0	0	0	0	0	−4
Derv 15	−4	−2	0	0	0	−2	0
Derv 16	−4	−2	−1	0	−1	0	0
Derv 17	−5	−2	0	0	0	−3	0
Derv 18	−5	−2	0	0	0	0	−3
Derv 19	−6	−2	0	0	0	−4	0

Total	82	28	8	8	7	14	23
Percent	100	34	10	10	9	17	28

**Table 3:** Validation cohort subset: difference (manual score − automatic score) greater than two. Difference in component scores is also included. Total represents the sum of the absolute value of the differences.

Patient	Diff	Resp	Coag	Liver	CV	CNS	Renal
Val 1	13	3	2	2	3	3	0
Val 2	5	2	0	0	0	0	3
Val 3	5	0	0	2	0	3	0
Val 4	5	1	0	0	0	4	0
Val 5	4	1	0	0	0	3	0
Val 6	4	0	1	0	0	4	−1
Val 7	4	0	0	0	0	4	0
Val 8	3	3	0	0	0	0	0
Val 9	3	0	2	0	0	0	1
Val 10	3	0	0	0	0	0	3
Val 11	3	0	1	0	0	1	1
Val 12	3	0	0	0	0	3	0
Val 13	3	0	0	0	0	3	0
Val 14	3	0	0	0	0	3	0
Val 15	3	0	−1	0	0	4	0
Val 16	−3	0	0	0	0	−3	0
Val 17	−3	−1	0	0	0	−2	0
Val 18	−3	−3	0	0	0	0	0
Val 19	−3	0	0	−2	0	0	−1
Val 20	−3	0	0	0	0	0	−3
Val 21	−4	0	0	0	0	−4	0
Val 22	−4	0	0	0	0	0	−4
Val 23	−11	−3	−2	0	0	−3	−3

Total	98	17	9	6	3	47	20
Percent	100	17	9	6	3	48	20

**Table 4:** Real-time AWARE cohort: a three-point scaled scoring system is used for comparison of the manual and automatic SOFA scores.

		Organ system components scores					
		Resp	Coag	Liver	CV	CNS	Renal
Difference (manual − automatic)	2	12	0	0	1	0	0
	1	20	0	1	3	0	0
	0	22	60	59	55	52	55
	−1	6	0	0	0	8	3
	−2	0	0	0	1	0	2
